# Neonatal Parotitis: A Case Report

**DOI:** 10.5811/cpcem.2021.3.51501

**Published:** 2021-05-06

**Authors:** Ayush Gupta, Tyler Kingdon, Andrew McKernan

**Affiliations:** * Children’s Hospital Of New Orleans, Department of Pediatric Emergency Medicine, New Orleans, Louisiana; † Louisiana State University Health Sciences Center, Department of Pediatrics, New Orleans, Louisiana

**Keywords:** Parotitis, neonate, purulent

## Abstract

**Introduction:**

Acute suppurative parotitis is a rare finding in the neonate. It is commonly caused by *Staphylococcus aureus*, but other bacterial isolates may be emerging. It is a novel disease for this age group and requires unique management. Only 32 cases of neonatal suppurative parotitis have been described in the English-language literature over the last 35 years.

**Case Report:**

We describe a case of a 14-day-old male who presented to the pediatric emergency department with a 24-hour history of swelling and redness of the right cheek. On examining him, purulent material was seen inside his oral cavity. He was subsequently hospitalized with a diagnosis of neonatal suppurative parotitis and received five days of parenteral antibiotics with improvement in swelling and redness. He was discharged home with oral antibiotics.

**Conclusion:**

Although neonatal suppurative parotitis is rare, it should be suspected in newborns presenting with an erythematous pre-auricular mass with or without any predisposing factors. We describe a rare case of acute suppurative parotitis in a neonate and review the published literature.

## INTRODUCTION

Acute suppurative parotitis is a rare finding in the neonate. It is commonly caused by *Staphylococcus aureus*, but other bacterial isolates may be emerging. It is a novel disease for this age group and requires unique management. We describe a case and present a literature review of neonatal parotitis based on a 14-day-old male who presented to the pediatric emergency department (ED) with swelling and redness of the right cheek and was diagnosed with acute suppurative parotitis.

## CASE REPORT

A 14-day-old male presented to the ED with a 24-hour history of swelling and redness of the right cheek. He had been doing well since birth. His mother noticed that he was crying between his feeds and appeared fussier. The parents denied fever, any kind of rash, or contact with a sick person. The cheek swelling was associated with the skin redness. The baby was born at 38 weeks twin delivery, cesarean section, no complications at birth, and was gaining weight appropriately.

**Image 1:**
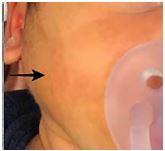
Black arrow shows swelling and erythema over the right side of the face in neonate with suppurative parotitis.

In the ED, the patient was afebrile with normal vitals for his age. On exam, he had right facial redness and crying with palpation of the right cheek (Image 1). The left side was normal. The right tympanic membrane could not be visualized due to edema. Oral mucosa was normal, but purulent material was seen coming out of the mouth. When parotid massage was performed more purulent material was expressed inside the mouth from the opening of the right Stensen duct. No other significant exam findings were noted. A presumptive diagnosis of acute suppurative parotitis was made.

**Image 2:**
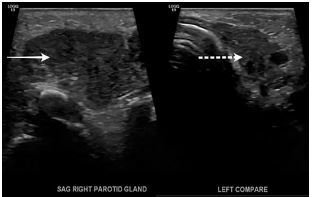
Parotid point-of-care ultrasound: swollen right-side parotid gland (solid white arrow) compared to the left-side parotid gland (dotted white arrow).

**Image 3:**
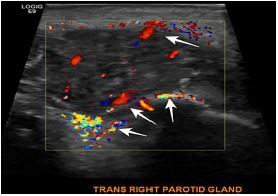
Parotid point-of-care ultrasound with Doppler showing increased vascularity (multiple white arrows) around the swollen right parotid gland, confirming parotitis.

His laboratory tests showed a normal complete blood count. His comprehensive metabolic panel was also within normal limits except for total bilirubin and direct bilirubin levels (Table 1). A respiratory panel was obtained and was negative. A point-of-care ultrasound of the soft tissue, head, and neck was performed, which showed a swollen, hypervascular right parotid gland likely representing changes of parotitis. There was no evidence of ductal dilation, obvious calcification shadowing, or changes to suggest abscess (Images 2 and 3). This was the main contribution to the diagnosis. Ear nose throat (ENT) and infectious disease specialists were consulted from the ED and agreed with the diagnosis. Even though the baby was afebrile, he received a full septic work-up. Because neonatal suppurative parotitis (NSP) causes complications such as bacteremia and meningitis in many cases, it needs to be identified and recognized early. Blood, urine, and cerebrospinal fluid (CSF) analysis was done (Table 2).Meningitis BioFire polymerase chain reaction panel (BioFire Diagnostics, Salt Lake City, UT) was negative. Urinalysis was a catheterized specimen that showed 2+ blood likely due to trauma associated with catheterization but no other findings. Cerebrospinal fluid analysis was normal. Intravenous (IV) antibiotics vancomycin, cefepime, and metronidazole were started to cover staphylococcal, streptococcal, and anaerobic species commonly responsible for causing acute suppurative parotitis. In the hospital, ENT recommended warm compresses and massage of the parotid gland that briefly expressed purulent drainage. However, cultures were not taken due to lack of availability of staff and minor duration of purulent drainage. Blood and CSF cultures did not show any growth, but urine cultures grew 10,000 colony forming units per milliliter *S. aureus*. After 48 hours of IV antimicrobials rapid clinical improvement was noticed. After five days of IV antibiotics, he was transitioned to oral clindamycin for 10 days with a resolution. He was sent home and the parents were advised to follow up with his pediatrician.

**CPC-EM Capsule**

What do we already know about this clinical entity?

*Neonatal suppurative parotitis is a rare condition. Diagnosis is made clinically with purulent material exuded from the Stensen duct being a pathognomonic sign.*

What makes this presentation of disease reportable?

*This patient presented with purulent fluid coming out of his mouth and redness of face. Use of point-of-care ultrasound (POCUS) with clinical findings, prompted early laboratory testing and antibiotics.*

What is the major learning point?

*Use of POCUS and cultures from purulent fluid help in diagnosis and need for full sepsis workup as suppurative parotitis can lead to bacteremia and meningitis.*

How might this improve emergency medicine practice?

*Neonatal suppurative parotitis is an uncommon infectious presentation without fever needing prompt recognition and management.*

## DISCUSSION

Neonatal suppurative parotitis is an uncommon disease, with a prevalence of 3.8:10,000 of neonatal admissions in an Italian study by Speigel et al in 2004.^1^ The same study reviewed all papers published in the English since 1970, reporting 32 cases.^1^ Decembrino et al^2^ reported 16 more cases of NSP described since 2004.

The parotid gland is more frequently infected than the other salivary glands because of its exclusive serous secretion without the bacteriostatic properties of the mucoid component.^2^ Although acute parotitis may affect normal healthy neonates, it seems to be more common in premature infants with low birth weight.^3^–^5^ This is presumptively related to a higher risk for dehydration, which may reduce salivary secretion causing salivary stasis. This stasis promotes bacterial ascent along the salivary duct.^2^ Bacterial seeding of the parotid gland can also occur hematogenously.^3^ Other risk factors implicated in parotitis are nasogastric intubation, sepsis, structural glandular abnormalities, cephalic or facial trauma, and immunodeficiency/immunosuppression.^3^ Transient immunoglobulin A deficiency is found in neonates, which affects mucociliary clearance and can lead to head and neck infections including rhinosinusitis, otitis media, mastoiditis, adenotonsillitis, and parotitis.^6^

*Staphylococcus aureus* is the most common pathogen isolated. Less-frequent agents are other Gram-positive cocci *(Streptococcus pyogenes, Streptococcus agalactiae, Streptococcus viridans)*, Gram-negative bacilli *(Escherichia coli, Klebsiella pneumoniae, Pseudomonas aeruginosa)*, and rarely anaerobic agents *(*Peptostreptococcus species*, Bacteroides melaninogenicus, Fusobacterium nucleatum,* Prevotella species*).*^2^,^4^–^7^ Although theoretically anaerobes are far more common in the normal oral flora, outnumbering aerobes by 10–100 times,^8^ most cases are still caused by *S. aureus.*

The diagnosis is based on clinical findings. In most cases NSP was unilateral and the most prevalent sign at the time of admission was swelling with or without redness of the parotid region. Fever is found in less than half of the cases. Bacteremia is present in up to 90% of cases and there can be meningitis associated with 33%. Purulent material exuded from Stensen’s duct is a pathognomonic sign of NSP.^1^,^9^

The differential diagnosis includes infectious and non-infectious etiologies. Infectious causes include lymphadenitis, cellulitis, soft-tissue abscess, osteomyelitis, buccinator muscle infection, and parotitis.^2^,^3^,^10^,^11^ Cellulitis-adenitis syndrome represents another possible focal manifestation of late-onset group B Streptococcus (GBS) infection, which is more common than parotitis, with an incidence estimated at 4%.^7^ Similarly to NSP, it presents with inflammatory signs typically involving unilateral facial or submandibular regions, which can be hard to distinguish from parotitis.^11^,^12^ Non-infectious etiologies of NSP include facial trauma, subcutaneous fat necrosis, and tumors such as lymphangiomas, hemangiomas, lipomas, or adenomas.^2^–^5^

Ultrasound findings can confirm the diagnosis, as in our case. Ultrasound can also be useful to identify soft tissue abscesses requiring surgical intervention, or non-infectious etiologies.^13^ Laboratory results are usually nonspecific, with leukocytosis and neutrophilia the most predominant findings. The serum amylase level is elevated in only a few cases, probably due to the immaturity of this salivary isoenzyme activity in newborns, rendering it unhelpful to the diagnosis.^1^ Cultures obtained from blood and purulent material from Stensen’s duct are essential for accurate diagnosis and therapy guidance. Lumbar puncture for CSF analysis should ideally be performed to safely determine adequate antibiotic therapy and its duration, as well as for outcome and follow-up purposes.^9^,^10^,^13^

The diagnosis of NSP in our patient was made based on clinical findings and with ultrasonography confirmation. After collecting the blood cultures, urine cultures, and CSF cultures, empiric IV antibiotic therapy with vancomycin and cefepime was immediately started. This covered the most common pathogens such as *S. aureus*, other Gram-positives such as GBS, and Gram-negatives. Based on the possible anaerobic causes, metronidazole was added. Most authors recommend starting therapy with an association of an anti-staphylococcal agent and an effective antibiotic against Gram-negatives, with adjustments after the results from the cultures are available.^1^,^2^,^5^,^10^, The increase of methicillin-resistant staphylococci may require the use of vancomycin. In the presence of an anaerobic cause, the treatment may include a combination of metronidazole and a macrolide or penicillin and β-lactamase inhibitor (clavulanate).^3^ A period of 7–10 days of this therapy appears to be adequate^2^,^3^ and generally leads to clinical improvement within the first 48 hours.

Most cases are managed conservatively, with prompt antibiotic therapy and adequate hydration essential for a good outcome. Surgical intervention is reserved for the rare cases with an inadequate response to medical therapy or for those with organized abscesses.^13^,^14^ The infection has a good prognosis, rare recurrence, and complications (facial palsy, fistula, mediastinitis and extension to the external auditory canal).^14^

## CONCLUSION

Although neonatal suppurative parotitis is rare, it should be suspected in newborns presenting with an erythematous pre-auricular mass with or without any predisposing factors. *S. aureus* is the most common pathogen isolated from infants with NSP. Exudate culture should be sent for testing. Most patients can be treated conservatively, provided that the empiric antibiotic treatment covers the causative agents according to the local sensitivity pattern and is started early. The prognosis of the disease is generally excellent.

## Figures and Tables

**Table 1:** Laboratory test results for neonate with suppurative parotitis.

	Patient’s results	Reference range
White blood cell	16,630 /μL	5,000–20,000 /μL
Neutrophils	56% (↑)	25–55%
Hemoglobin	13.5 g/dL	12.9–20.5 g/dL
Hematocrit	38%	39–59%
Platelets	450,000 /μL	150,000–450,000 /μL
Total bilirubin	16.1 mg/dL (↑)	0.3–1.0 mg/dL
Direct bilirubin	0.7 mg/dL (↓)	> 2 mg /dL
C-reactive protein	0.6 mg/dL	< 1mg /dL
Procalcitonin	0.17 ng/mL	< 0.5ng /ml

*μL*, microliter; *g*, gram; *dL*, deciliter; *mg*, milligram; *ng*, nanogram.

**Table 2:** Results of cerebrospinal fluid analysis shows traumatic lumbar puncture.

	Patient’s results	Reference range
Glucose	46 mg/dL	38–175 mg/dL
Protein	142 mg/dL	29–160 mg/dL
Red blood cells	7,301 /μL (↑)	0–236 /μL
White blood cells	0 /μL	0–13 /μL

*μL*, microliters; *dL*, deciliters; *mg*, milligrams.
